# The Role of Epigenetics and Contributing Impact of Stress, Multigenerational, and Developmental Factors in Opiate Addiction

**DOI:** 10.7759/cureus.53788

**Published:** 2024-02-07

**Authors:** Jason Do

**Affiliations:** 1 Physical Medicine and Rehabilitation, State University of New York Downstate Health Sciences University, Brooklyn, USA

**Keywords:** opioid use, bench work, opiates, addiction, epigenetic modification

## Abstract

Drug addiction is characterized by maladaptive neural plasticity, particularly in vulnerable individuals exposed to drugs of abuse. Epigenetic factors include environmental influences, events during development, and stress adaptations, which seem to play an important role in the neuropathogenesis of drug addiction. This critical review hypothesizes that epigenetic modulation increases an individual’s susceptibility to opiate addiction in three key areas of epigenetic study: developmental, stress-related, and transgenerational effects.

The widespread use of opioids for clinical and recreational purposes raises significant societal and scientific concerns. Despite the increasing prevalence of opioid abuse, there is limited comprehensive knowledge about the impact of epigenetic factors on opiate addiction manifestation. This review hypothesizes that epigenetic modulation increases susceptibility to opiate addiction, exploring three key areas of epigenetic study: developmental, stress-related, and transgenerational effects.

Current literature reveals a correlation between epigenetic influences and vulnerability to drug addiction, specifically in the context of opioid use. Epigenetics, the modulation of genetic expression beyond genotypic predisposition, plays a crucial role in an individual’s susceptibility to drug addiction. Studies suggest that epigenetic mechanisms, once considered static in the adult brain, continue to influence synaptic plasticity and long-term memory, particularly in the endogenous opioid system.

This review examines the effects of opioids and stress on epigenetic modifications, providing evidence of increased vulnerability to opiate addiction. Animal studies demonstrate how developmental adversities and adolescent exposure to substances can induce persistent epigenetic changes, predisposing individuals to opiate addiction in adulthood. Moreover, the review explores the transgenerational effects of opioid exposure during adolescence, suggesting that functional epigenetic neuroadaptations within the nucleus accumbens can persist for multiple generations. The examination of DNA methylation patterns in opioid addicts reveals potential markers for identifying susceptibility to opiate vulnerability.

A critical analysis of research reports supports the hypothesis that developmental, transgenerational, and stress-related epigenetic mechanisms have a profound role in increasing the risk of opioid addiction susceptibility. Each study confirmed that developmental, stress-related, or transgenerational epigenetic regulations have a correlation to increased opiate sensitization and vulnerability. Unfortunately, every study reviewed was unable to elucidate an epigenetic mechanism to explain a specific neuropathogenesis of opiate drug addiction vulnerability, emphasizing our lack of knowledge in the complex pathology of epigenetics.

## Introduction and background

Drug addiction can be described as a maladaptive neural plasticity that occurs in vulnerable individuals in response to repeated exposure to a drug of abuse [1]. Opioid addiction is prevalent in society causing both short-term physical dependence and long-term vulnerabilities mediated by alterations in the mesocorticolimbic dopamine system [1]. This poses the question: what are the existential factors that influence “vulnerability” to drug addiction? Besides the evidence that genetic disposition contributes about 50-60% to an individual’s “addictive vulnerability,” modulation of genetic expression has recently been found to offer great insights into our understanding of one’s susceptibility to drug addiction. Such epigenetic factors include environmental influences, events during development, and stress adaptations, which seem to play an important role in the neuropathogenesis of drug addiction.

The abundant use of opioids for clinical as well as recreational purposes makes it an area of crucial societal as well as scientific interest. To further understand the consequences of opioid use, it is important to investigate factors that may impact vulnerability to opiate addiction. As a relatively new field of study, there is a paucity of comprehensive knowledge regarding the effects of epigenetic factors on the manifestation of opiate addiction. Ultimately, this critical review hypothesizes that epigenetic modulation increases an individual’s susceptibility to opiate addiction in three key areas of epigenetic study: developmental, stress-related, and transgenerational effects.

Background

Considering the effectiveness and widespread use of opioid analgesics, it is not surprising that the abuse of and addiction to opioids has become a prominent global issue. In 2021, the United States was estimated to have 2.7 million people suffering from prescribed opioid use disorder [2]. In fact, global opioid use has shown a marked increase as opioids persist as the group of substances most significantly associated with drug-related harm. Approximately 60 million individuals were involved in non-medical opioid usage in 2021, with 31.5 million predominantly using opiates, particularly heroin [2,3]. Recent surveys have found that exposure to opiate prescription use has increased in adolescent populations that are still at a developmental stage of maturation, affecting their vulnerability to drug addiction. This poses a complex problem for society, i.e., to balance our confrontational stance against opioid abuse while preserving the clinical benefits of opioids to reduce patient suffering. The current status of treatment focuses on symptom management as there is still no cure for drug addiction. Even if treatment is successful, there is still a life-long struggle with the possibility of relapse.

Our current knowledge of drug addiction etiology suggests a correlation between epigenetic influences on the vulnerability and relapse of drug addiction. Epigenetics addresses the mechanisms that promote phenotypic changes in response to factors beyond our genotypic predisposition. More specifically, the term epigenetics refers to the modulation of genetic expression through the manipulation of DNA/chromatin activity. It also connotes the transgenerational heritability of these mechanisms and their influence on phenotypic plasticity [4]. As mentioned before, an individual’s susceptibility to drug addiction is affected by both genetic and epigenetic factors. We have some understanding of typical epigenetic regulation of transcriptional mechanisms, particularly through the modification of histone proteins or DNA itself. These genetic modifications were initially thought to be “statically quiet in the adult brain” [5]. However, recent studies have found that these epigenetic mechanisms may play an ongoing role in synaptic plasticity and long-term memory, especially in the communication system mediated by endogenous opioid peptides [5]. Endorphins, enkephalins, dynorphins, and endomorphins are endogenous opioid peptides that originate from their precursors, pro-opiomelanocortin, proenkephalin, and prodynorphin, to induce activity via opioid receptors. Previous studies have shown that opioid receptor alleles have characteristics that make them more susceptible to epigenetic regulation, e.g., genes that are rich in CpG islands: regions that contain cytosine-guanine dinucleotides where cytosine is susceptible to methylation [6]. This enables modification of promoter regions as well as chromatin remodeling that modulates expression [6]. Continuing this line of study, it may be possible to determine how environmental, developmental, stressor, or transgenerational factors explain today’s “risk factors” and even preventive or clinical intervention for opiate addiction. However, epigenetic study of the opiate system is relatively new and it may be a while before we have the means to elucidate addictive vulnerability through epigenetics.

Nevertheless, our efforts to understand the role of epigenetics in the pathogenesis of opiate addiction promise greater insights into an individual’s vulnerability to opiate drug addiction.

Statement of methods

A comprehensive search of basic and clinical research reports in PubMed and UNODC world drug reports over the past two decades provided the foundation for this critical review of epigenetic mechanisms that may impact the onset of opiate addiction. The review focuses on three key epigenetic dimensions: developmental, stressors (especially in early life), and transgenerational factors. To determine the extent to which current research supports or opposes the hypothesis, this review analyzes the hypotheses and results of relevant experiments, as well as their experimental limitations.

## Review

Developmental epigenetic induction of opiate use vulnerability

Epigenetic effects during development give us an idea of how early ontogenetic influences can shape adult phenotypes. Inimical environmental stressors (physical and emotional) may alter the developmental mechanisms that enhance vulnerability to opioid addiction. Although there are many studies that support a strong correlation between adverse life experiences and drug addiction, the evidence to identify a causal relationship between the two is lacking in a human model. If it can be shown that adverse events during development produce epigenetic modifications clearly related to opioid use, it may be possible to establish a necessary causal relationship that will enable rational intervention.

Previous studies have shown that the separation of newborn pups from their mother predisposed rats to anxiety and opioid intake during adolescence and adulthood. Having a diminished maternal presence was reported to alter the hypothalamic-pituitary-adrenal (HPA) function due to epigenetic mechanisms affecting glucocorticoid expression [7]. Therefore, Tesone-Coelho et al. conducted a study to determine the causal effects of maternal deprivation on vulnerability to opioid intake through particular epigenetic mechanisms [8]. They focused particularly on the methyl-CpG-binding protein, MeCP2, a transcriptional repressor that inhibits gene expression after binding to methylated DNA and consequently recruits histone deacetylases (HDACs). It yields an inactive chromatin state and silences downstream genetic expression. MeCP2 has a role in the normal development of the central nervous system, modulating synaptic transmission by regulation of spontaneous neurotransmission and short-term synaptic plasticity. HDACs (primarily HDAC2) are believed to play an important role in cognitive function, inducing memory deficits when overexpressed. Thus, Tesone-Coelho et al. hypothesized that by inducing developmental modifications via maternal deprivation in newborn rats, MeCP2 would be elevated in the striatum, and HDAC2 expression and activity would increase in the nucleus accumbens due to decreased acetylation of histones H3 and H4. They used sodium valproate, an HDAC inhibitor found to restore acetylation of histones, and found that it blocked the escalating consumption of morphine in rats.

Ultimately, their experiment compared two groups of rats: maternally deprived (D) rats and a control group with maternal presence (AFR). The D rats experienced three hours of daily isolation for the first 14 days of life. On day 22, they were weaned and housed until three months of age when they were sacrificed to evaluate the epigenetic markers MeCP2, HDAC2, HDAC3 and acetylated histones H3 and H4. The experiment effectively identified the presence of epigenetic regulation (explained next), as maternal deprivation was associated with increased opioid consumption.

The study supported their hypothesis that the increased percentage of MeCP2-expressing cells was not only apparent at the age of 14 days but stayed elevated until adulthood at 90 days old. In 90-day-old rats, MeCP2 expression was elevated throughout the striatal dopaminergic projections but not in the ventral tegmental area (VTA) where these neurons originate. There were elevated levels of MeCP2 in the caudate-putamen (CPu), and both the shell (NAcSh) and core (NAcCo) regions of the nucleus accumbens. Considering the molecular complex that MeCP2 often makes with class I HDACs, they measured HDAC2 and HDAC3 expression as well as acetylation of H3 and H4 to determine HDAC enzyme activity. Comparing the D and AFR groups, they only found statistically significant changes for HDAC2 immuno-reactive cells in the CPu and NAcCo; HDAC3 had no significant changes. This is important to note, considering that the CPu and NAcCo were the only two areas measured that showed a significant decrease of acetylated H3 and H4 proteins. This indicates that early life isolation can have a profound effect on epigenetic regulation in areas that are known for their role in reward/reinforcement and drug addiction.

To determine the extent to which elevated HDAC activity due to maternal deprivation affected the oral morphine consumption rate, Tesone-Coelho et al. examined the effects of sodium valproate, an HDAC inhibitor. Their results were consistent with their hypothesis, as the oral morphine consumption was significantly increased only in D rats. Rats treated with sodium valproate experienced temporary weight loss that recovered and exhibited morphine consumption and preference similar to the AFR control group. Lastly, they conducted a western blot analysis demonstrating that the attenuation of oral morphine consumption by sodium valproate was correlated with HDAC activity on H3 and H4 proteins. This indicates that increased HDAC activity due to elevated MeCP2 in D rats correlates to an increased rate of morphine consumption. This experiment demonstrates that the increased opioid consumption was due to HDAC activity: an epigenetic mechanism.

These results support the assertion that maternal deprivation during development increases vulnerability to opioid consumption. Tesone-Coelho et al. showed in the rat model that epigenetic mechanisms involving MeCP2 expression and consequent HDAC activation enhance vulnerability to opioid drug addiction. This conclusion is significant, as it exemplifies how adverse postnatal influences (viz., bonding) during rat development can play a direct role in promoting opioid consumption later in life. Furthermore, the elevated presence of epigenetic markers supports the hypothesis that epigenetic activity contributes to this susceptibility to increased opioid consumption.

Tesone-Coelho et al. confirmed that sodium valproate treatment normalized morphine consumption rates and abolished the increase of nuclear HDAC activity in the nucleus accumbens (NAc) of group D rats while halting the reduction of H4 acetylation. Through the removal of an acetyl functional group from key histone residues, HDACs promote an inactive chromatin state, silencing downstream gene expression. Class I HDACs, a family that is recruited by MeCP2, a global transcriptional repressor, play a role in synapse maturation and function. While the rat model is convenient and can often be related to human pathology, there are obvious limitations for studies of brain function, as the human brain is far more developed than that of the rat.

Increased expression of HDAC2 is known to impair memory formation as it silences gene expression related to synaptic plasticity and neuronal activity [9]. However, the specificity of numerous HDACs for specific lysine residues remains a mystery as hundreds of proteins are thought to be recruited to a gene in concert with activation or repression, ultimately emphasizing the complexity of epigenetic HDAC mechanisms [1]. The causative relationship remains to be elucidated. Considering that sodium valproate is known to act on aspartate and γ-amino butyric acid neurotransmission, its attenuating mechanism in oral morphine consumption is still unclear [10]. The Tesone-Coelho et al. experimental data strongly suggest that developmental epigenetic mechanisms influence susceptibility to opiate addiction. However, it is difficult to propose a concrete neurobiological mechanism due to the lack of knowledge about epigenetic mechanistic interactions. To elucidate the causative relationship between maternal deprivation and increased MeCP2, as well as the neuropathology of increased opioid consumption and HDAC activity, further studies could examine the effects of maternal deprivation on different epigenetic regulatory pathways, e.g., histone methylation, acetylation, different HDAC classes, etc. [11].

In addition to adverse social-environmental impacts on development, exposure to other drugs and chemicals during adolescence may have an impact on adult opioid vulnerability. Tomasiewicz et al. investigated the effects of cannabis exposure in adolescent rats on adult heroin self-administration [12]. Investigating the “gateway drug” stereotype of cannabis, this study offers insights into the neuropathological correlation of its potential enhancement of opiate addiction. Previous studies have reported that adolescent exposure to Δ9-tetrahydrocannabinol (THC) has profound effects in altering the enkephalinergic opioid system by increasing the expression of the endogenous opioid peptide precursor, proenkephalin (Penk) in the NAcSh [13,14]. In response, Tomasiewicz et al. used viral-mediated gene transfer strategies to determine whether the opiate addictive behavior is produced by the overexpression of Penk in response to adolescent THC exposure.

Using a lentiviral vector encoding Penk and green fluorescent protein (GFP), Tomasiewicz et al. created three rat groups: a GFP-control group, a Penk-infused group, and a group of THC-exposed adolescents that resulted in Penk overexpression. To determine whether Penk overexpression facilitated a susceptibility to opioid abuse, they used two behavioral assessments: a cue-induced reinstatement assessment and stress-induced drug-seeking enhancement. By associating heroin delivery with responding on a drug-paired lever, they measured the cue-induced response to the drug-related stimuli after cessation. Their data revealed that THC-exposed adolescents and NAcSh Penk-overexpressed rats showed a statistically significant increase in drug-paired lever response, unlike the GFP-control group. One week later, they subjected all three groups to a stressor (24-hour food deprivation) to determine if it elicited enhanced drug-seeking behavior. Again, both THC-exposed adolescents and the NAcSh Penk groups showed an increased propensity for heroin-seeking behavior after the stress-inducing event. To confirm whether this behavior was due to the presence of Penk, they utilized microRNA (miR)-mediated mRNA cleavage specific to NAcSh Penk mRNA to knock down its expression. Decreased NAcSh Penk levels were associated with significantly diminished heroin intake and reduced drug-seeking behavior. Lastly, they examined histone H3 methylation at the Penk gene in the NAcSh. Of all the specific histone H3 methyl marks observed, only H3K9me3 and H3K4me3 at the Penk gene showed elevated levels in adulthood. Although these findings are impressive, further study of H3K9me3 and H3K4me3 could tell us how developmental epigenetic modulation contributes to opiate sensitivity phenotypes in adulthood.

The Tomasiewicz et al. experiments support their hypothesis that THC increases Penk expression, which suggests an impact on opiate addictive behavior. By testing Penk-infused rats separately, the experiment suggests the change in drug-seeking behavior is due to the presence of this opioid peptide precursor as opposed to another systemic effect of THC. Additionally, the miR-mediated knockdown of Penk mRNA demonstrated the attenuation of opioid addictive behavior. Epigenetic modulation from THC exposure has a significant implication: the resulting NAcSh Penk overexpression induces a vulnerability to increased heroin self-administrative behavior. This study demonstrates this correlation well by identifying concurrent behavioral influences in the GFP-control, Penk-infused, and THC-exposed rat models. Ultimately, they are successful in evaluating how developmental epigenetic modulation can predispose adult opiate addictive behaviors.

The Tomasiewicz et al. experimental model has a very narrow scope and is unable to explicate the neuropathogenesis of opiate addiction vulnerability. Their emphasis on Penk creates the assumption that Penk is the predominant endogenous opioid peptide precursor to impact opiate addiction. However, our current understanding of the neuropathology is still not complete. There is no established causal mechanism between NAcSh Penk activity and the induction of opioid addiction. Additionally, this study focuses entirely on the self-administration assessment models. While it is effective in determining influences on intake and behavioral responses, the experimental model does not disentangle reward and incentive motivational states, which have a major role in shaping the foundation of drug addiction. Therefore, future studies are needed to elucidate these developmental epigenetic mechanisms and confirm their impact on the reward pathways that induce adult vulnerability to opioid addiction.

Cannabis exposure may potentially have epigenetic effects before the developmental period of adolescence. DiNieri et al. conducted a study to determine whether maternal use of cannabis during prenatal development impacts NAc dopamine subtype 2 (D2) gene regulation in offspring [15]. The D2 receptor is also known to express the endogenous opioid peptide enkephalin [16]. In addition, previous studies have shown that the D2 receptor is a consistently downregulated characteristic in adult drug addiction [17]. Therefore, the D2 receptor is a notable point of interest to determine if diminished D2 receptor expression plays a role in opiate addiction vulnerability. DiNieri et al. investigated striatal dopamine and opioid-related genes in aborted human fetal subjects that were exposed to cannabis, cigarettes, and alcohol. Furthermore, any cannabis-related influences found in human fetuses were further studied using an animal model of prenatal THC exposure. This study hypothesized that maternal cannabis use induced fetal epigenetic downregulation of D2 receptors to predispose the offspring to opiate addiction.

In the human fetuses exposed to prenatal cannabis use, there was a significant decrease of detected D2 receptors in the human fetal striatum (NAc and putamen). This reveals a negative correlation between the maternal use of cannabis and striatal levels of D2 receptors in the offspring. In comparison, the control vehicle (VEH) had no impact on striatal D2 receptor levels. Although they assessed the levels of Penk and prodynorphin (PDYN) in the human fetal striatum to account for the generalized effects of maternal cannabis use, there were no statistically significant differences between VEH and cannabis-exposed fetuses. One caveat of using an unregulated human fetus sample size is the unreliability of self-reporting. Nevertheless, in response to the fetal subject results, DiNieri et al. investigated whether THC exposure reduced D2 receptor expression and its impact in a rat model.

As suspected, the rat model paralleled the results found in the human fetus. Prenatal exposure to THC induced a 40% decrease in DR2 receptor mRNA expression in rat NAc measured by in situ hybridization histochemistry. With an established reduction in D2 receptor levels, the study conducted chromatin immunoprecipitation on NAc extracts of adult male rats prenatally exposed to THC and immunoprecipitated with antibodies specific to dimethylated lysine 9 (2meH3K9) and trimethylated lysine 4 (3meH3K4) [15]. DiNieri et al. studied these acknowledged modifications because the decreased D2 receptor expression could be modulated by either transcriptional repression (2meH3K9) or activation (3meH3K4). After immunoprecipitation, they found that prenatal THC exposure led to a significant increase in the repressive 2meH3K9 park and a decrease in 3meH3K4 in the NAc of adult rats. This indicates that prenatal exposure to THC utilizes epigenetic regulatory mechanisms that repress D2 receptor expression.

Lastly, to determine whether decreased D2 receptor levels from prenatal exposure to THC influenced opiate addiction vulnerability, DiNieri et al. used morphine place conditioning to observe sensitivity to the rewarding effects of morphine. Results indicated that the adult rats exposed to prenatal THC had a significantly increased preference for the morphine-associated side of the compartment. Increased place preference in a conditioning paradigm indicates that decreased D2 receptor expression and prenatal THC exposure contribute to opiate reward sensitivity in adulthood.

DiNieri et al. had a very effective experimental progression. After identifying the effects of prenatal THC exposure in human fetuses, the study induced the same phenomenon in a rat model and confirmed the utilization of repressive epigenetic modifications that decreased D2 receptor expression. Although DiNieri et al. successfully identified the epigenetic marks that impact D2 receptor repression, further study of the mechanism would explain the neuropathology of how THC modulates 3meH3K4 or 2meH3K9 on the D2 receptor gene. The study utilized place preference conditioning to demonstrate the extent to which decreased D2 receptor levels from prenatal THC exposure elevate morphine sensitivity. As we know, many epigenetic modifications potentially modulate opioid addiction susceptibility. Further studies should investigate additional factors that modulate prenatal development or impact biological systems that alter opioid reward circuitry, e.g., the HPA axis.

Stress as a risk factor for opioid substance use disorders

Varghese et al. defined early life stress (ELS) to consist of a “wide spectrum of adverse experiences: physical, sexual, and emotional abuse; marital discord; parental loss; parental neglect; witnessing domestic violence; parental alcoholism; or living with those who have substance use disorders (SUD), or mental illness” [18]. While there are many potential causes of ELS, they all have a commonality affecting neuropathology related to drug addiction: the HPA axis. In response to stressors, corticotropin-releasing factor (CRF) triggers the HPA axis. Increased expression of CRF due to stressors modifies epigenetic regulation of neuronal mechanisms that drive reward or compulsivity pathways in opioid drug addiction. CRF activity is mediated by two receptor pathways: CRFR1 and CRFR2, having a higher non-selective affinity for CRFR1 which is the primary molecular site for stress activation [18]. Furthermore, previous studies report that endogenous opioid systems in the amygdala and NAc mediate opiate reward behavior [17]. Alcoholism in humans activates the endogenous opioid system by upregulating PDYN activity on kappa-opioid receptors (KORs) [19]. This implies that long-term upregulation of PDYN expression in the amygdala and NAc potentially impacts the vulnerability of opioid addiction [20].

Varghese et al. examined morphine sensitization and withdrawal symptoms in CRF-overexpressing (CRF-OE) mice and evaluated the PDYN expression in the amygdala and NAc of the CRF-OE mice treated with morphine. The study hypothesized that the overexpression of CRF in mice would upregulate PDYN expression and consequently elevate opioid drug addiction susceptibility.

They conducted two experiments to measure morphine sensitization and withdrawal symptoms in CRF-OE and WT (control) mice. The first experiment investigated morphine sensitization, measured as locomotor activity following repeated injections of morphine or saline. Morphine sensitization is a commonly used assessment to evaluate locomotor activity after the administration of psychotropic drugs [21]. An increase in morphine sensitization in mice leads to an enhanced desire for morphine itself and associated cues due to amplified incentive salience. Varghese et al. found significant differences between CRF-OE vs. WT mice in locomotor activity in trials four and five. Differentially greater morphine-induced locomotor sensitization among CRF-OE mice suggests higher sensitivity to opioids in this group.

The second experiment paralleled the drug intake pattern of humans with an opioid SUD. The CRF-OE and WT mice were first treated with increasing doses of morphine, and the investigators measured behavioral signs of withdrawal after morphine discontinuation. Opiate withdrawal was measured using four signs: the number of rearings, circling behavior, grooming, and jumps. Comparing the global score of all four signs of withdrawal, CRF-OE mice had a significantly higher score than that of WT mice. Morphine induced a stereotyped behavior prominently seen by the CRF-OE mice that demonstrated an increased circling behavior and number of jumps and a decrease in grooming and rearing. These results suggest that morphine withdrawal symptoms were elevated due to CRF overexpression in mice. Lastly, PDYN immunohistochemistry in the amygdala and NAc illustrated an overall increase in PDYN expression in CRF-OE mice.

In accordance with the Varghese et al. hypothesis, CRF overexpression in the forebrain was associated with increased locomotor sensitization and withdrawal symptoms in morphine-treated mice, as well as elevated PDYN protein expression in their amygdala and NAc. These results indicate that ELS-induced epigenetic modulations altered the typical regulation of CRF. Prior studies have shown that brain stress systems contribute to the three stages of the addiction cycle related to compulsivity: binge/intoxication, withdrawal/negative effects, and preoccupation/anticipation [22]. Exposure to ELS instigates epigenetic modifications that result in elevated levels of CRF and PDYN, as well as increased sensitivity to opioid use.

ELS potentially may alter neurodevelopment through interactions between epigenetic regulation mechanisms and physiological/mental stressors. However, the Varghese et al. data only offer a superficial probe into this phenomenon and does not explain the causative impacts of stressors. In the second experiment, the four different opioid withdrawal stereotypic actions are effective for the basic overview of the influence of CRF overexpression through neuroadaptive processes. However, each withdrawal behavioral sign may have different causative factors. Despite a lack of mechanistic understanding, the two Varghese et al. experiments only stressed the overall importance of CRF and its correlation to opioid sensitization and withdrawal symptoms.

As mentioned in the Varghese et al. study, CRF is the main neuropeptide released upon activation of the brain stress systems and contributes to behavioral and autonomic responses. We next examine the downstream effects of corticotropin-releasing factor receptors 1 and 2 (CRFR1/CRFR2). Both CRFR1 and CRFR2 are expressed in the dopaminergic neurons of the VTA and NAc, two areas implicated in the drug reward circuit [23]. Lasheras et al. researched the role of CRFR1 in the rewarding effects of opioids using a conditioned place preference (CPP) paradigm [23]. The hypothesis was that morphine-induced CPP is regulated by CRFR1 and that antagonizing this receptor would block morphine-induced CPP.

The CPP paradigm was used to evaluate the rewarding effects of morphine. Afterward, these two groups were separated into different compartments, where they were treated with an injection of morphine/CRF1R antagonist (CP-154,526) or saline/vehicle (Tween 80 10%). Lastly, they sacrificed the mice and used immunohistochemical detection of c-Fos/TH expression as an index of neuronal activation within dopaminergic neurons of the VTA and NAc.

From this experiment, they found that CP-154,526 significantly blocked the morphine-induced CPP and increased the corticosterone secretion response. These results indicate that although the CRFR1 antagonist blocked drug-paired place preference, it did not block the HPA axis activation instigated by morphine administration. Using elevated expression of c-Fos/TH to assess the brain reward systems of the VTA and NAc, they found that CP-154,526 significantly blocked the activation of the dopaminergic neurons in both brain regions. This experimental model effectively evaluates the relationship between CRFR1 and morphine sensitivity. Using the CPP paradigm as an opioid-related stimulus, they demonstrated a strong correlation between CRFR1 activity and opioid addiction neuropathology. Furthermore, c-Fos/TH labeling indexed dopaminergic neuronal activity in the brain reward circuit areas: the VTA and NAc. When the drug-paired mice were treated with the CRFR1 antagonist, c-Fos/TH levels were diminished in these key areas, indicating decreased reward activity from morphine treatment. Although the VTA and NAc are known areas of the reward circuit, further studies into the systemic or neurobiological impact of CRF could elucidate the broad study of stress epigenetic effects.

Overall, this study indicated that CRFR1 plays a critical role in the brain reward circuit induced by morphine-associated stimuli. Although there is a lack of knowledge about the mechanisms and roles of stress systems in the early phases of drug addiction, the Lasherasa et al. study confirmed the profound impact that CRF pathways have on the onset of opioid-addictive behaviors [18,23].

Multigenerational epigenetic effects of parental opioid drug exposure

In previous studies on the effects of morphine during the development of the adolescent female rat, exposed females and their future offspring demonstrated long-term alterations in µ- and κ-opioid receptors in the mediobasal hypothalamus [24]. The effects seen in their offspring cannot be attributed to direct exposure, ruling morphine exposure out, as both parents were abstinent from drug use at least three weeks before mating. This implied that the transgenerational epigenetic effects of morphine occurred before mating. Therefore, we can conclude that parental history of drug exposure can potentially influence the vulnerability to opioid addiction of their offspring. Byrnes et al. reported that first-generation male offspring of morphine-exposed mothers (MORF1) showed greater locomotor sensitization in response to repeated doses of morphine compared to the male offspring of saline-exposed mothers (SALF1) [25]. This cross-sensitization between morphine use and locomotion indicated that MORF1 rats are more responsive to opioid use. This is notable because it implies that opiate addiction vulnerability may be due to the persistent influence of epigenetic modulation. Byrnes et al. used another locomotor sensitization model to validate their observations in the first. Here, they tested the rat in response to the dopamine agonist: quinpirole, a D2/D3 dopamine receptor agonist that activates post-synaptic D2/D3 receptors in the NAc. The enhancement of D2/D3 activity in the NAc stimulates corticosterone secretion, providing an index of NAc function while revealing an interaction with stress mechanisms.

Byrnes et al. also conducted this study in such a way as to detect any transgenerational impact from opioid exposure during a female rat’s developmental period. Beginning in rat adolescence (30 days old), one group of females was injected with increasing doses of morphine for 10 days (MORF0), whereas the control group was treated with a saline equivalent (SALF0). Between three and four weeks after their last injection, MORF0 and SALF0 were mated with drug-free males to form progeny lines MORF1 and SALF1. Afterward, MORF1 and SALF1 lines were mated again with drug-free males to form a second generation: MORF2 and SALF2. Using these lines, adult F1 and F2-generation males were assessed using the quinpirole-challenge model to determine if there is a transgenerational influence on processes mediated by D2-like receptors in the NAc. Investigating the acute and repeated response to quinpirole injection, Byrnes et al. monitored rat locomotor behavior, viz., offspring D2/D3 receptor activity. In this study, they conducted a concurrent corticosterone analysis using a standard radioimmunoassay and quantitative polymerase chain reaction to measure D2 receptor and KOR expression.

While there were no differences based on maternal history for acute quinpirole treatment, repeated quinpirole treatment had significant differences compared to saline treatment. Examination of the locomotor activity results in the repeated quinpirole challenge in the F1 generation reveals an increased locomotor response to quinpirole over eight days of exposure. The F2 generation demonstrated similar increased locomotor activity in response to quinpirole. Although the quinpirole-treated rats had higher basal rates of locomotor activity, MORF1 and MORF2 displayed attenuated locomotor activity compared to their SALF1 and SALF2 counterparts. The authors reported that the difference in locomotor activity between MORF1 and SALF1 is due to the activation of “release-regulating presynaptic D2 receptors,” which results in less dopamine release in the NAc [25]. These results indicate that, despite quinpirole action as a D2/D3 receptor agonist, MORF1 and MORF2 generations inherited this trait as a result of MORF0 epigenetic regulation of D2 receptors. The blunted NAc dopamine release demonstrated by the MORF subjects appears to impact the reward circuitry, potentially contributing to predisposition to future opioid use.

In the corticosterone analysis, there were no significant differences between the SALF and MORF lines following acute quinpirole treatment. However, with repeated administration of quinpirole, there was an elevation of plasma corticosterone in both MORF1 and MORF2 males. As stated earlier, quinpirole is known to upregulate corticosterone production due to increased D2/D3 activity in the NAc. Similar to the repeated opioid use seen in human SUD, these results suggest repeated exposure to morphine stimulates D2/D3 dopaminergic activity in the reward pathway and influences the HPA axis. However, as these relationships are correlative, the mechanisms remain to be elucidated.

Lastly, repeated exposure to quinpirole increased D2R and KOR mRNA expression. The significant difference in D2R and KOR mRNA expression is attributed to the differing maternal history between the MORF and SALF generations. This data suggests the increased expression of NAc D2R and KOR mRNA seen in MORF1 and MORF2 is a consequence of opioid exposure during the adolescent development of MORF0. The upregulation of D2R and KOR expression is important to note considering their associated activity during opioid drug exposure. The upregulation of D2R expression can be due to fewer D2 receptors, a trait often seen in drug abusers that may suggest a predisposition to opioid use [20]. KOR activation in the NAc inhibits D1R-mediated activity, a key mechanism in the development and expression of locomotor sensitization, as well as localization in the striatonigral pathway that employs the opioid peptide Dynorphin [15]. Therefore, increased KOR activation in MORF generations may explain the attenuated locomotor activity during repeated exposure to quinpirole and the potential vulnerability to opioid use.

Byrnes et al. results are especially noteworthy because they demonstrate transgenerational consequences of adolescent morphine exposure in systems that regulate reward and stress, presumably mediated by epigenetic modification. The most prominent findings of this study are the persisting effects of increased D2R and KOR expression found in the F2 generation. Thus, epigenetic modifications are conserved across at least two generations, emphasizing the persistent effects of opioid drug addiction. These findings support the Byrnes et al. hypothesis that female opioid exposure serves as a multigenerational influence on neurodevelopment, increasing the risk and vulnerability to mental health, stress-related disorders, and opioid substance abuse.

The Byrnes et al. studies used a valid and reliable experimental model to determine the occurrence of transgenerational epigenetic effects: observing the differences between the SALF and MORF lines as well as saline- and quinpirole-treated rats. Although the data support the Byrnes et al. hypothesis, there are notable limitations. Through the action of D2 receptors in the NAc, quinpirole also induced an increase in corticosterone secretion seen in MORF1 and MORF2. The measure of corticosterone was used primarily as an indirect measure of D2 receptor activity. However, as noted by Varghese et al., stress mechanisms themselves play a role in the susceptibility of opioid sensitization. Glucocorticoid levels alone do not explain the neuropathology of opiate addiction vulnerability, emphasizing the importance of understanding stress-mediated signaling molecule interactions [26].

As the mechanism for multigenerational epigenetic inheritance is unknown, there are still possibilities of factors other than NAc D2R affecting the F1 and F2 generations, e.g., possible drug effects on F0 maternal gametes, behavioral change that alters social-environmental development of their offspring, or prenatal influence on early neurodevelopment [27]. Continued study of the transgenerational epigenetic mechanism may offer insights into specific factors that induce susceptibility to opioid addiction in offspring.

Considering the clinical implications of these observations, male opioid addicts may be subjected to epigenetic modification that can impact their future offspring. To study this possibility, Chorbov et al. conducted a study to determine whether hypermethylation of blood DNA (blood cells) in the OPRM1 promoter region is associated with opioid addiction and whether these effects are also present in sperm DNA [28]. OPRM1 refers to the gene that encodes for the µ-opioid receptor (MOR), a common target for frequently used opioids, such as heroin, morphine, and methadone [28]. Importantly, OPRM1 includes CpG sites that are prone to DNA methylation.

Comparing blood and semen samples from recovering opiate addicts and a control group, Chorbov et al. examined the average levels of DNA methylation over the whole CpG island. Between opiate addicts and the control group, the average experimental results only indicated a significant difference in blood DNA. Analysis of single CpG sites revealed that significant hypermethylation occurred at seven sites in blood DNA from addicts. In sperm-derived DNA from addicts, the level of methylation only increased significantly at one CpG site. The methylated CpG sites in these subjects underwent epigenetic modulation that upregulates the OPRM1 gene expression. Despite the marginal difference across the entire CpG Island, the DNA methylation percentages demonstrate that sperm DNA from opiate addicts is consistently higher than the control group. Considering that the difference between addict and control sperm DNA methylation is not statistically significant, it is still unclear whether opiate addict sperm DNA contributes to the transgenerational vulnerability of opiate addiction.

In response to the Chorbov et al. hypothesis, the results support the claim that increased DNA methylation in the OPRM1 gene is associated with opioid addiction, demonstrated by the hypermethylated CpG sites found in opiate addict blood DNA. These CpG sites will potentially block transcription activators that would lead to OPRM1 silencing and therefore downregulation of MOR. This study has a very narrow focus on a human sample group that consequently includes many variations between subjects. To continue the study on male gamete transgenerational epigenetics, an isolated animal model will serve better to eliminate exogenous factors and permit focused characterization of points of interest.

Discussion

An overall critical analysis of these primary research findings supports the hypothesis that developmental, stress-related, and transgenerational epigenetic modifications induce an increased vulnerability to the onset of opioid drug addiction. One may find that these developmental and transgenerational events are contributory to one’s genetic predisposition to opioids and possibly substance abuse in general. While these three risk factors may seem isolated, the epigenetic inductions are a multifactorial cascade of mechanisms that ultimately lead to a common pathology and disposition. By the accumulation of stressors in life, development, or familial history, one may find themselves more likely to succumb to opioid addiction.

The Tesone-Coelho et al. study demonstrated that maternal deprivation induced a predisposition to opioid intake through the modification of certain epigenetic parameters in the striatum: increased MeCP2 expression and HDAC activation. This emphasized the sensitivity of the developmental period to social and environmental influences. The elevated MeCP2 expression in response to maternal deprivation in adolescence was reported to be long-lasting, remaining at a comparatively high level for 10 weeks after the last maternal deprivation [8]. This claim suggests that the persistence of epigenetic modulations materialized from adverse developmental life events. If human subjects react similarly to developmental adversities such as maternal deprivation, it would not be surprising if they displayed epigenetic modifications that parallel elevated MeCP2 expression and produced a predisposition to opiate addiction in adulthood.

Tomasiewicz et al. demonstrated that THC-exposed adolescent rats upregulated NAcSh Penk, which consequently increased heroin self-administration. One epigenetic modification that is potentially responsible for the upregulation of Penk in the NAcSh is the reduction of H3K9 di-methylation and tri-methylation, both of which are involved in active gene regulation [12]. The persistent effects of the epigenetic dysregulation of Penk may explain THC’s long-term effects on opiate sensitivity. As adolescent exposure to THC upregulated Penk and increased opioid consumption, cannabis could provide an epigenetic “gateway” to opiate addiction vulnerability.

DiNieri et al. investigated the effects of cannabis exposure in prenatal developmental periods. Their results suggested that prenatal cannabis exposure disrupts developmental epigenetic regulation to reduce D2 receptor expression in the NAc. Given that D2 receptor dysregulation is implicated in addiction risk, prenatal THC exposure may contribute to opiate addiction onset if it diminishes adult D2 receptor levels. The profound effects of THC exposure emphasize the sensitivity of the prenatal developmental period to environmental influences. It is important to note that the dysregulation of D2 receptor expression was attributed to prenatal THC disrupting long-term epigenetic regulation. Events that impact long-term epigenetic mechanisms during development may potentially explain the persistent effects of opioid drug vulnerability.

Animal models have demonstrated how chronic stress can lead to increased drug self-administration due to structural plasticity changes in the mesolimbic reward system [8,27]. Varghese et al. demonstrated a correlation between CRF mechanisms and addiction-related phenotypes. CRF expression following morphine treatment leads to elevated PDYN protein expression in brain regions relevant to addiction (amygdala and NAc). This suggests that ELS events that influence long-term CRF expression may contribute to the etiology of drug addiction. However, given our understanding of stress mechanisms in the brain, it is still unclear how stressors may alter gene expression. For instance, corticosterone injections did not produce the same expression profile as acute stress, which implies that in vivo stressors may have activity through diverse pathways outside the glucocorticoid system [26].

Lasherasa et al. furthered our study of CRF interactions by investigating the role of CRF1R in response to morphine rewards in a CPP paradigm. CRF1R was found to play a pivotal role in morphine-mediated responses. The study reported that morphine-related behaviors were attenuated after CRF1R antagonist administration, suggesting CRF1R has a direct correlation to addictive behaviors. The persistent effects of ELS may be explained by a stress-related epigenetic modulation resulting in the persistent activation of CRF1R to promote adulthood opioid sensitivity and eventually addiction.

The research of Byrnes et al. demonstrated the transgenerational effects of adolescent morphine exposure in systems that regulate reward and stress. Quinpirole treatment attenuated sensitization to locomotor effects, increased corticosterone secretion, and increased NAc expression of KOR and D2 mRNA. These findings suggest that functional epigenetic neuroadaptations within the NAc can persist for two generations following F0 generation adolescent opioid exposure. This is notable because it implies opiate vulnerability is multigenerational. Emphasizing the sensitivity of developmental epigenetic regulation, opioid exposure during a female’s adolescence maintains a persistent effect not only for her lifetime but for at least two future generations. If we were able to identify a similar mechanism in humans, we may be able to gain a greater understanding of the impact family history may have on opioid SUD as well as other inheritable neurological disorders.

Lastly, Chorbov et al. investigated whether male opioid addicts had elevated levels of DNA methylation at the OPRM1 promoter. This study was able to demonstrate that opiate addicts can be identified by elevated levels of DNA methylation of the OPRM1 promoter in blood DNA. However, levels of DNA methylation of OPRM1 in opiate addicts were not statistically significant from controls, making it unclear whether male opiate addicts predispose their offspring to opiate vulnerability. The marginal difference of methylation between addict and control sperm DNA may provide the foundation for further investigation of the etiology of transgenerational epigenetic regulation on male gametal DNA and its effects on offspring opioid vulnerability.

## Conclusions

Epigenetic research provides insights into how social-environmental factors, experiences, and stressors shape phenotypic expression. A critical analysis of research reports supports the hypothesis that developmental, transgenerational, and stress-related epigenetic mechanisms have a profound role in increasing the risk of opioid addiction susceptibility. Each article confirmed that developmental, stress-related, or transgenerational epigenetic regulations have a correlation to increased opiate sensitization and vulnerability. Unfortunately, every study reviewed was unable to elucidate an epigenetic mechanism to explain the neuropathogenesis of opiate drug addiction vulnerability, emphasizing our lack of knowledge in the complex pathology of epigenetics. In our journey to clarify the primary pathways associated with early life development, this review allows us insights into the possible management and etiology of drug addiction.

Clinically, it is important to improve our understanding of epigenetic mechanisms to identify possible risk factors in the development of opioid SUDs. As emphasized throughout the critical review, there is a broad spectrum of epigenetic factors that could potentially prompt a life-long opioid vulnerability. The significant increase in the adolescent use of prescription opioids only promotes the urgency to study the developmental effects of epigenetic factors and stressors. Through a better understanding of the neuropathology of epigenetics on opiate addiction, we gain a better understanding of preventative treatment and clinical risk factors.
